# Machine Learning–Based Prognostic Model for Patients After Lung Transplantation

**DOI:** 10.1001/jamanetworkopen.2023.12022

**Published:** 2023-05-05

**Authors:** Dong Tian, Hao-Ji Yan, Heng Huang, Yu-Jie Zuo, Ming-Zhao Liu, Jin Zhao, Bo Wu, Ling-Zhi Shi, Jing-Yu Chen

**Affiliations:** 1 Department of Thoracic Surgery, West China Hospital, Sichuan University, Chengdu, China; 2 Wuxi Lung Transplant Center, Wuxi People’s Hospital affiliated to Nanjing Medical University, Wuxi, China; 3 Department of General Thoracic Surgery, Juntendo University School of Medicine, Tokyo, Japan; 4 Department of Clinical Medicine, North Sichuan Medical College, Nanchong, China

## Abstract

**Importance:**

Although numerous prognostic factors have been found for patients after lung transplantation (LTx) over the years, an accurate prognostic tool for LTx recipients remains unavailable.

**Objective:**

To develop and validate a prognostic model for predicting overall survival in patients after LTx using random survival forests (RSF), a machine learning algorithm.

**Design, Setting, and Participants:**

This retrospective prognostic study included patients who underwent LTx between January 2017 and December 2020. The LTx recipients were randomly assigned to training and test sets in accordance with a ratio of 7:3. Feature selection was performed using variable importance with bootstrapping resampling. The prognostic model was fitted using the RSF algorithm, and a Cox regression model was set as a benchmark. The integrated area under the curve (iAUC) and integrated Brier score (iBS) were applied to assess model performance in the test set. Data were analyzed from January 2017 to December 2019.

**Main Outcomes And Measures:**

Overall survival in patients after LTx.

**Results:**

A total of 504 patients were eligible for this study, consisting of 353 patients in the training set (mean [SD] age, 55.03 [12.78] years; 235 [66.6%] male patients) and 151 patients in the test set (mean [SD] age, 56.79 [10.95] years; 99 [65.6%] male patients). According to the variable importance of each factor, 16 were selected for the final RSF model, and postoperative extracorporeal membrane oxygenation time was identified as the most valuable factor. The RSF model had excellent performance with an iAUC of 0.879 (95% CI, 0.832-0.921) and an iBS of 0.130 (95% CI, 0.106-0.154). The Cox regression model fitted by the same modeling factors to the RSF model was significantly inferior to the RSF model with an iAUC of 0.658 (95% CI, 0.572-0.747; P < .001) and an iBS of 0.205 (95% CI, 0.176-0.233; P < .001). According to the RSF model predictions, the patients after LTx were stratified into 2 prognostic groups displaying significant difference, with mean overall survival of 52.91 months (95% CI, 48.51-57.32) and 14.83 months (95% CI, 9.44-20.22; log-rank *P* < .001), respectively.

**Conclusions and relevance:**

In this prognostic study, the findings first demonstrated that RSF could provide more accurate overall survival prediction and remarkable prognostic stratification than the Cox regression model for patients after LTx.

## Key Points

**Question:**

Does the random survival forests (RSF) model provide a personalized and accurate prediction for overall survival in patients after lung transplantation?

**Findings:**

In this prognostic study of 504 patients after lung transplantation, the RSF model had excellent performance with an integrated area under the curve of 0.879 and an integrated Brier score of 0.130.

**Meaning:**

In this study, the RSF showed promising results for predicting the overall survival of patients after lung transplantation.

## Teaser

This prognostic study evaluates a machine learning–based prognostic model for predicting the survival of patients after lung transplantation.

## Introduction

Lung transplantation (LTx) provides improved survival and quality of life to patients with end-stage lung diseases. Despite advances in surgical techniques and perioperative management, the posttransplant survival outcome is still unsatisfactory with a median survival of 6.7 years in adult patients after LTx.^1^ Personalized and accurate survival prediction can help clinical decision-making and further improve the posttransplant survival of patients after LTx. Many studies have reported numerous independent prognostic factors related to donors, recipients, surgery, complications, hematology, and radiology.^2,3,4^ However, these factors are challenging to use in accurately predicting survival outcomes or are difficult to apply in clinical practice. More importantly, it is troublesome for lung transplant surgeons to integrate many factors to judge a precise prognosis.

A prediction model developed by traditional regression analysis or a machine learning approach could integrate a mass of prognostic parameters and provide individual survival prediction.^5^ Reports about prognostic models for LTx recipients are rare, and models have delivered unsatisfactory performance in predicting the survival outcome of patients.^6,7,8,9^ Therefore, a personalized and well-performing prognostic tool is necessary for patients after LTx, although one is not yet available. Random survival forests (RSF), as part of machine learning algorithms, are designed to be used specifically for survival outcome prediction and have shown promising performance in our previous study.^10^ The RSF could build a mass of decision trees with a log-rank test to identify different survival statuses and produce an individual probability derived from the average prediction results across all trees.^11^ Compared with conventional regression analysis, RSF takes advantage of freedom from application restrictions and excellent prognostic performance. However, the RSF algorithm has not yet been studied in patients after LTx. This study aimed to develop and test a prognostic model based on the RSF algorithm for predicting overall survival (OS) in patients after LTx and to compare its performance with a benchmark model fitted using Cox regression.

## Methods

### Patients

Patients who underwent LTx at Wuxi People’s Hospital between January 2017 and December 2019 were reviewed. Adult patients (>18 years) with complete follow-up records who underwent LTx were enrolled in this prognostic study (eFigure 1 in Supplement 1). Of the patients who had LTx, 6 patients with retransplant, 7 with a pediatric lung transplant, and 6 with severe missing data were excluded from this study. Then, patients were randomly subclassified into a training set and a test set at a ratio of 7:3 for subsequent analysis. The ethics committees and review board of the Affiliated Hospital of Nanjing Medical University approved the current prognostic study, and informed consent was waived due to the nature of the retrospective study. This study adhered to the Transparent Reporting of a Multivariable Prediction Model for Individual Prognosis or Diagnosis (TRIPOD) reporting guidelinereporting guideline.^12^

### Data Collection

Medical records were reviewed to collect the clinical characteristics and survival statuses of patients after LTx. A total of 22 characteristics, consisting of 4 recipient factors, 1 donor factor, 4 transplant procedural factors, and 13 posttransplant factors were collected. OS was the target variable in the current study. The follow-up interval after LTx for judging survival status was 3 to 5 weeks, and the last follow-up in this study occurred in February 2022. The missing data were handled by multiple imputation by chained equations and performed by “mice” package in R.

### Statistical Analysis

#### Model Development

All analyses in this study were realized using R version 4.2.1 (R Project for Statistical Computing). The feature selection procedure was performed according to variable importance (VIMP), an internal statistic of the RSF algorithm as our previous study described.^10^ The bootstrapping resampling method with 1000 repetitions was used to increase the robustness and calculate the 95% CI of VIMP. Factors with a mean VIMP greater than 0.01 were included in the final RSF model. The grid search method was used for hyperparameter tuning.

The RSF model was fitted using the “randomForestSRC” R package to predict the OS of patients after LTx according to the selected factors and the optimal hyperparameter combination.^13^ We also developed a Cox regression model based on the same factors as a benchmark using the “rms” R package.^14^ These prediction models can identify linear or nonlinear relationships between characteristics and survival outcomes, and provide a predicted value to achieve the outcome prediction for a new sample.

#### Model Validation

We validated the model performance using the hold-out method, which indicates testing the model using the data in the test set. For further validation of the generalization capacity of the model, the test set was categorized according to the surgical type (single LTx [SLTx] and double LTx [DLTx]) and diagnosis (interstitial pulmonary fibrosis [IPF] and chronic obstructive pulmonary diseases [COPD]). In the absence of any specific statement to the contrary, all performance statistics were calculated according to the entire test set.

Model performance was assessed in 2 aspects: discrimination and calibration. The integrated area under the curve (iAUC) and the time-dependent area under the curve (tAUC) were used to evaluate the model’s discrimination ability. The integrated Brier score (iBS) and the prediction error (PE) were applied to estimate the calibration ability. The differences between model’s performance were calculated using a bootstrapping method with 1000 repetitions. If the 95% CI of the differences did not cover a 0 value, the differences were considered statistically significant. A 2-sided *P* value less than .05 was considered statistically significant. The “cutp” function in the “survMisc” R package was used to divide patients into 2 prognostic categories according to the predicted value.^15^ Meanwhile, we also divided patients into 3 categories according to the 33.3% and 66.7% quantiles. More descriptions of methods can be found in eMethods in Supplement 1. Data were analyzed from January 2017 to December 2019.

## Results

### Characteristics of Patients

We eventually included 504 patients after LTx with a mean (SD) age of 55.56 (12.27) years; 334 were male (66.3%). Most patients were diagnosed with interstitial pulmonary fibrosis (IPF) (275 patients [54.6%]), which was followed by chronic obstructive pulmonary disease (COPD) (98 patients [19.4%]) as the next most common diagnosis. There was a similar number of patients receiving single lung transplantation (SLTx) and double lung transplantation (DLTx), (236 [46.8%] and 268 [53.2%], respectively). The patients were randomly grouped into the training set (353 patients) and test set (151 patients). We summarized the detailed characteristics of 3 sets of patients in Table 1. The mean (SD) follow-up time in this cohort was 35 (19) months. For all patients after LTx in this study, the 1-year, 3-year, and 5-year survival rates were 64.9%, 55.5%, and 48.8%, respectively (eTable 1 in Supplement 1).

**Table 1.** Clinical Characteristics of Patients After Lung Transplantation

Characteristics	Patients, No. (%)		
	All patients (N = 504)	Training set (n = 353)	Test set (n = 151)
Age, mean (SD), y	55.56 (12.27)	55.03 (12.78)	56.79 (10.95)
Sex			
Male	334 (66.3)	235 (66.6)	99 (65.6)
Female	170 (33.7)	118 (33.4)	52 (34.4)
Body mass index, mean (SD)^a^	20.54 (3.52)	20.33 (3.54)	21.02 (3.42)
Diagnosis			
Interstitial pulmonary fibrosis	275 (54.6)	191 (54.1)	84 (55.6)
Chronic obstructive pulmonary diseases	98 (19.4)	67 (19.0)	31 (20.5)
Pulmonary arterial hypertension	15 (3.0)	13 (3.7)	2 (1.3)
Pneumoconiosis	61 (12.1)	44 (12.5)	17 (11.3)
Others	55 (10.9)	38 (10.7)	17 (11.3)
Surgical approach			
Lateral sequential	401 (79.5)	278 (78.7)	123 (81.5)
Supine sequential	94 (18.7)	67 (19.0)	27 (17.9)
Clamshell	9 (1.8)	8 (2.3)	1 (0.6)
Surgical type			
Single lung transplantation	236 (46.8)	164 (46.5)	72 (47.7)
Double lung transplantation	268 (53.2)	189 (53.5)	79 (52.3)
ECMO type			
Venovenous	233 (46.2)	155 (43.9)	78 (51.7)
Venoarterial	131 (26.0)	103 (29.2)	28 (18.5)
None	140 (27.8)	95 (26.9)	45 (29.8)
72 h PGD3			
No	381 (75.6)	262 (74.2)	119 (78.8)
Yes	123 (24.4)	91 (25.8)	32 (21.2)
Preoperative hormone use			
No	293 (58.1)	207 (58.6)	86 (57.0)
Yes	211 (41.9)	146 (41.4)	65 (43.0)
Multidrug-resistant bacterial infection			
No	46 (9.1)	35 (10.0)	11 (7.3)
Yes	458 (90.9)	318 (90.0)	140 (92.7)
Operation time, mean (SD), min	336.5 (99.41)	338.75 (98.33)	331.24 (102.01)
Postoperative ECMO time, mean (SD), d	1.38 (2.13)	1.44 (2.12)	1.24 (2.14)
Postoperative ventilator time, mean (SD), d	5.78 (12.9)	5.87 (12.74)	5.56 (13.31)
ICU stay, mean (SD), d	7.5 (9.59)	7.62 (9.84)	7.22 (8.99)
6MWT, mean (SD), m	462.62 (77.54)	465.73 (79.13)	455.33 (73.42)
Cold-ischemia time, mean (SD), h	7.58 (2.02)	7.59 (1.98)	7.55 (2.14)
Donor Pao_2_/Fio_2_, mean (SD)	440.43 (70.63)	440.82 (69.85)	439.52 (72.63)
FEV1, mean (SD)	2.04 (0.54)	2.06 (0.56)	2.01 (0.48)
FEV1%, mean (SD)	0.67 (0.16)	0.67 (0.16)	0.68 (0.14)
FVC, mean (SD)	2.49 (0.63)	2.51 (0.65)	2.42 (0.57)
FVC%, mean (SD)	0.66 (0.15)	0.66 (0.15)	0.66 (0.13)
FEV1/FVC, mean (SD)	0.83 (0.1)	0.82 (0.1)	0.84 (0.08)

Abbreviations: ECMO, extracorporeal membrane oxygenation; FEV1, forced expiratory volume in 1 second; FEV1%, percentage of predicted forced expiratory volume in 1-second value; FVC, forced vital capacity; FVC%, percentage of predicted forced vital capacity value; ICU, intensive care unit; Pao_2_/Fio_2_, arterial oxygen tension/inspired oxygen fraction; 6MWT, 6-minute walking test; 72 h PGD3, grade 3 primary graft dysfunction at 72h.

^a^ Body mass index is calculated as weight in kilograms divided by height in meters squared.

### Model Development

**Figure 1.**
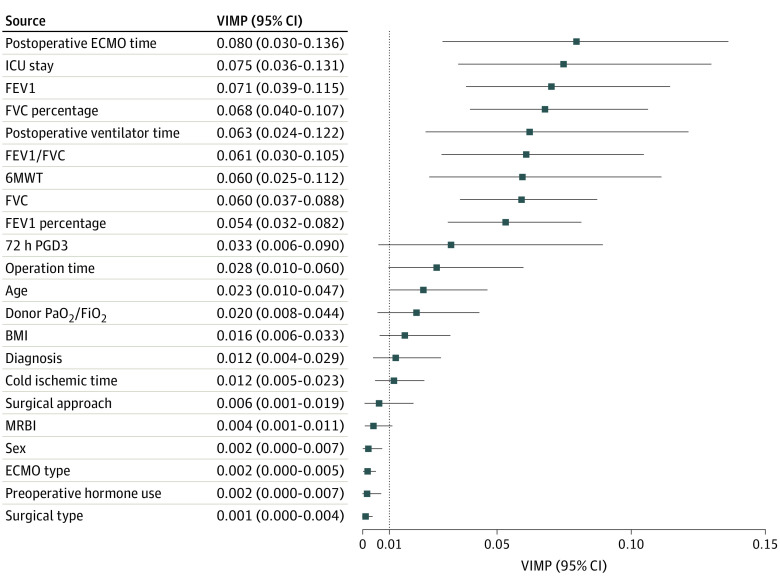
Variable Importance of the Candidate Characteristics in RSF Model In this figure, the VIMP was validated by the bootstrapping method with 1000 repetitions and shown as a mean with 95% CI. The top 15 features were included in the final RSF model. Abbreviations: BMI, body mass index; ECMO, extracorporeal membrane oxygenation; FEV1, forced expiratory volume in 1 second; FEV1 percentage, percentage of predicted forced expiratory volume in 1-second value; FVC, forced vital capacity; FVC percentage, percentage of predicted forced vital capacity value; ICU, intensive care unit; MRBI, multidrug-resistant bacterial infection; Pao_2_/Fio_2_, arterial oxygen tension/inspired oxygen fraction; RSF, random survival forests; VIMP, variable importance; 6MWT, 6-minute walking test; 72 h PGD3, grade 3 primary graft dysfunction at 72h.

Out of 22 factors, 16 had a VIMP greater than 0.01 validated by the bootstrap method, and these variables were selected for the final RSF model (Figure 1). Meanwhile, the postoperative extracorporeal membrane oxygenation (ECMO) time was determined to be the most crucial factor for the RSF model, with a bootstrapped VIMP of 0.080 (95% CI, 0.030-0.136).

### Overall Survival Prediction

In terms of OS prediction, the RSF model showed excellent discrimination (iAUC of 0.879; 95% CI, 0.832-0.921) as well as calibration (iBS of 0.130 95% CI, 0.106-0.154) (Table 2). The predicted survival by the RSF model showed great agreement with the observed survival (eFigure 2 in Supplement 1). The performance of the Cox model was significantly inferior to that of the RSF model, with an iAUC of 0.658 (95% CI, 0.572-0.747; *P* < .001) and an iBS of 0.205 (95% CI, 0.176-0.233; *P* < .001). The RSF model consecutively outperformed the Cox regression model from 1 to 48 months in terms of discrimination and calibration (eFigure 3 in Supplement 1).

**Table 2.** The RSF Model and Conventional Cox Regression Model for Predicting Overall Survival in Patients After Lung Transplantation

Models	Time of prediction	iAUC/tAUC (95% CI)	*P* value^a^	iBS/PE (95% CI)	*P* value^a^
RSF model	1 to 48 mo	0.879 (0.832-0.921)	[Reference]	0.130 (0.106-0.154)	[Reference]
Cox model	1 to 48 mo	0.658 (0.572-0.747)	<.001	0.205 (0.176-0.233)	<.001
RSF model	1 mo	0.858 (0.792-0.917)	[Reference]	0.123 (0.096-0.153)	[Reference]
Cox model	1 mo	0.624 (0.523-0.728)	<.001	0.181 (0.100-0.219)	<.001
RSF model	1 y	0.921 (0.877-0.957)	[Reference]	0.115 (0.095-0.139)	[Reference]
Cox model	1 y	0.717 (0.633-0.800)	<.001	0.195 (0.098-0.225)	<.001

Abbreviations: Cox, Cox regression; iAUC, integrated area under the curve; iBS, integrated Brier score; PE, prediction error; tAUC, time-dependent area under the curve; RSF, random survival forests.

^a^ Comparison with the performance of Cox model to RSF model with the same time of prediction.

**Figure 2.**
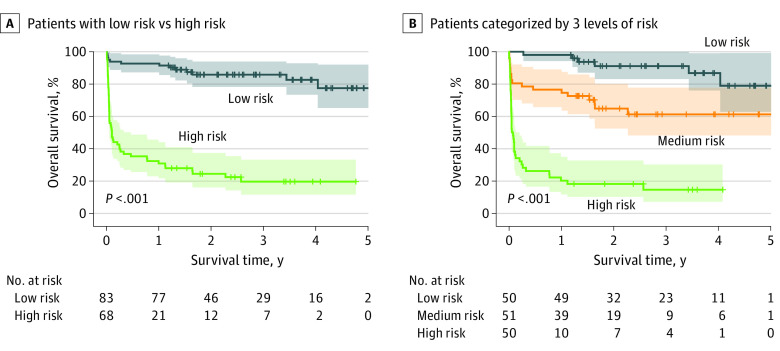
Survival Curves for Patients After LTx With Different Risks Stratified Using the RSF Model The RSF model divided patients after LTx in the test set to 2 categories (panel A) and 3 categories (panel B) with significant differences in overall survival (both *P* < .0001). LTx indicates lung transplantation; RSF, random survival forests.

According to the best survival difference, the optimal threshold of the predicted value by the RSF model was 30.74, and the patients in the test set were divided into low- and high-risk groups. The patients in the high-risk group displayed significantly worse survival than those in the low-risk group, with mean overall survival of 14.83 months (95% CI, 9.44-20.22) and 52.91 months (95% CI, 48.51-57.32; log-rank *P* < .001), respectively (Figure 2A). Furthermore, patients were categorized into low-, medium-, and high-risk groups as demarcated by threshold values of 16.33 (33.3% quantile) and 42.98 (66.7% quantile). The patients in the high-risk group had the worst survival, followed successively by those in the medium-risk and low-risk groups, with mean overall survival of 9.74 months (95% CI, 4.91-14.58), 41.01 months (95% CI, 33.31-48.71), and 54.58 months (95% CI, 50.17-59.00; log-rank *P* < .001), respectively. (Figure 2B).

### One-Month and 1-Year Survival Prediction

Although the discrimination for the survival prediction at 1 month slightly decreased with a tAUC of 0.858, the calibration was fairly good with a PE of 0.123. Moreover, the tAUC and PE of the RSF model for 1-year survival prediction were 0.921 and 0.115, respectively, which were greater than the values for OS prediction. Regardless of prediction for survival after LTx at 1 month or 1 year, the RSF model was better than the Cox model (Table 2).

**Figure 3.**
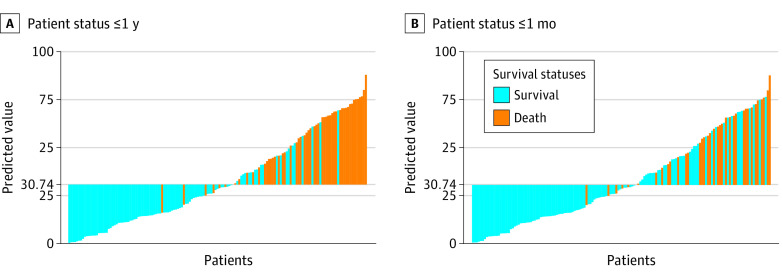
The Distribution of the Predicted Value by RSF Model The patients after lung transplantation with different statuses within 1 year (panel A) or 1 month (panel B) have disparate trends of the predicted value by RSF model (both *P* < .0001). RSF indicates random survival forests.

The patients who survived 1 month showed a significantly lower predicted value by the RSF model than those who died, with a mean difference of 29.61 (95% CI, 22.67-36.53; *P* < .001) (Figure 3A; eTable 2 in Supplement 1). The RSF model for 1-month survival prediction had a sensitivity of 86.1%, a specificity of 68.7%, and an accuracy of 72.9%. In addition, the patients who survived 1 year had a significantly lower predicted value than those who died (mean predicted value: 20.84 vs 55.45; *P* < .001) (Figure 3B; eTable 2 in Supplement 1). The RSF demonstrated good performance for 1-year survival prediction with a sensitivity of 88.7%, a specificity of 79.6%, and an accuracy of 82.8%.

### Model Testing in Subgroups

The performance of the RSF in specific subgroups is detailed in eTable 3. Both patients with SLTx or DLTx could be accurately predicted by the RSF model with an iAUC of 0.861 and 0.896, respectively. The iBS values of the RSF model in SLTx and DLTx were 0.159 and 0.096, respectively. The RSF model accurately predicted the survival of patients with IPF (iAUC, 0. 885; and iBS, 0.143). However, iAUC and iBS of RSF for patients with COPD were 0.809 and 0.150, respectively, which were general compared with other subgroups. Regardless of whether patients were diagnosed with SLTx (log-rank *P* < .001), DLTx (log-rank *P* < .001), IPF (log-rank *P* < .001), or COPD (log-rank *P* = .001), the high-risk group exhibited a significant difference in survival (eFigure 4 in Supplement 1).

## Discussion

The current study first applied a novel approach, the RSF model, to provide a personalized and accurate posttransplant survival prediction for patients after LTx. The highlighted findings of this study are as follows. First, the postoperative ECMO time was the most critical factor in predicting OS among the 22 clinical characteristics. Second, the performance of the conventional Cox regression model was significantly inferior to that of the RSF model in terms of discrimination and calibration. Third, the RSF model exhibited excellent performance in predicting the OS or specific time point (1-month and 1-year) survival outcomes for patients after LTx.

Since the introduction of ECMO, it has gradually become a versatile and crucial treatment strategy for LTx recipients. Postoperative ECMO use is widely known as one of the main prognostic factors for short- or long-term survival.^2,16^ ECMO can support LTx recipients with severe graft dysfunction after LTx and provide an improved survival outcome for patients.^17,18^ However, these patients have a worse prognosis than others due to their poor general condition, even when supported by ECMO. Recently, from the latest meta-analysis to date,^2^ which pooled 72 eligible studies, it was found that the posttransplant need for ECMO is the only prognostic factor with high certainty for 1-year mortality. Our results further confirmed the prognostic value of the posttransplant need for ECMO. More importantly, our findings confirmed the prognostic significance of posttransplant ECMO use from another perspective (using VIMP).

In the past decade, precise prognosis assessment has been pursued to optimize clinical decision-making for lung transplant surgeons. Nevertheless, a conventional approach based on prognostic factors to identify patient survival outcomes has some problems, such as insufficient accuracy for a single prognostic factor and difficult integration for many prognostic factors. The prognostic model, also known as the prediction model, is an effective method for generating a personalized prediction and provides a solution to these problems of the conventional approach.^19^ A well-validated prognostic model could integrate various prognostic factors and produce an individualized survival prediction. For patients who have undergone LTx, several reports have presented results of studying prognostic models to predict OS^6,9^ or survival at specific time points.^7,8,20^ However, these models showed a limited performance, which could not realize an accurate prediction. Moreover, no prognostic model based on posttransplant factors has been reported. In the current study, we fitted and tested an excellent prognostic model to predict posttransplant OS of patients after LTx with an iAUC of 0.879, and this model was effective in providing a personalized survival prediction. In addition, partial patients may not be able to receive lung function examination and the 6-minute walking test (6MWT), especially those who are dead within 1 month. Therefore, we have added 2 additional analyses, which excluded lung function/6MWT data or excluded patients who died within 1 month, to further assess the performance of the RSF model (eTable 4 in Supplement 1). The RSF model performed well in both conditions, with iAUC of 0.800 and 0.834, respectively. These results suggest that the RSF model can still provide a fairly accurate prediction even without some examination data. Our model showed promising results mainly because of the application of a suitable machine learning algorithm.

As a novel method to realize personalized risk evaluation, machine learning algorithms can learn the patterns of high-dimensional data of many patients and provide a personalized prediction.^5^ Although machine learning is promising in medical practice, few studies have reported its application in LTx.^21,22^ Recently, a study reported a random forests model to predict 1-year survival, but the AUC of this model is 0.62. To our knowledge, the current study first introduced the RSF algorithm into prognostic research in patients after LTx. At first, the RSF algorithm, derived from the random forests algorithm, is explicitly designed to be used for survival data.^23^ The RSF has an advantage over Cox regression in 2 aspects. First, the RSF can be used in high-dimensional data, but Cox regression is restricted by 2 assumptions: (1) hazard functions are proportional over time, and (2) the relationship between the hazard and covariates is linear.^24^ Second, the RSF model can explore the nonlinear relation between outcome and variables, but Cox regression only hunts for linear relation. The relation between variables and OS probably is nonlinear for patients after LTx, and that may be the reason why the RSF model showed a better performance. In this study, we confirmed the superiority of the RSF model for LTx recipients compared with the Cox model, consistent with previous studies.^25,26^ Moreover, we also tried stepwise selection to determine the modeling factors for the Cox regression model. However, its performance was still inferior to that of the RSF model (eTable 5 in Supplement 1). In addition, although this model outperformed the Cox model, the RSF algorithm, such as other machine learning algorithms, has a problem of interpretation.^27^ Therefore, VIMP was applied in this study not only to select features but also to make the RSF model more interpretable.

We conducted 2 additional tests to further validate the generalization capacity of the RSF model. First, in addition to OS, 2 significant time points related to LTx recipients (1-month survival and 1-year survival) are worth monitoring. The 1-month survival status represents the perioperative efficacy of LTx, and the 1-year survival status reflects the long-term prognosis. The median survival of LTx recipients would improve to 10.2 years conditional on survival to 1 year^2^. A specific logistic regression model may be established to predict 1-month or 1-year survival in previous studies.^28^ In our study, we found that the RSF model performed well in predicting survival at any time point, including 1 month and 1 year. This finding further demonstrated good prognostic value and extended the application of the RSF model. Second, we preliminarily tested the generalization capacity of the RSF model in 4 subgroups with different surgical types or diagnoses. Although patients with SLTx vs DLTx and IPF vs COPD have varied prognoses after LTx,^29^ the RSF model accurately predicted outcomes for patients with different traits. All results of subgroup validation suggested that the RSF model has excellent generalization capacity and potential to generalize to other LTx recipients.

### Limitations

The limitations of the current study are as follows. First, potential bias may exist due to the single-center retrospective study design. As a pilot study applying the RSF algorithm to LTx, our promising results signify that a prospective study with a large sample size is imperative. Second, our models were tested only by an internal test set. For a machine learning model, the test data from other centers are essential for examining generalization capacity. Therefore, a multicenter study is warranted to further confirm our findings. Third, this study included only 22 characteristics to develop the RSF model. Some potential factors that may be associated with the prognosis in patients after LTx were not included in this research. Fourth, we did not perform a separate analysis for pediatric patients after LTx. Considering the difference between adult and pediatric patients, whether the RSF model still performs well to predict survival in pediatric patients is undetermined. However, our center has an insufficient sample size for a separate analysis of pediatric patients, and further research is warranted.

## Conclusions

In this prognostic study, as a machine learning approach, the RSF model provided personalized and accurate survival prediction and remarkable prognostic stratification for patients after LTx. The RSF algorithm may outperform the traditional Cox regression in survival prediction for LTx recipients. This study first introduces the RSF model to prognostic surveillance of patients after LTx, and the proposed method may improve clinical decision-making for lung transplant surgeons.

## Notes

Supplement 1. **eMethods. **Additional Information About Data Collection, Model Development, and Model Validation **eTable 1. **Overall Survival Rate for Patients After Lung Transplantation **eTable 2. **Predicted Value by RSF Model in Patients With Different Survival Statuses **eTable 3. **Subgroup Tests for the RSF Model **eTable 4. **The Performance of RSF Model in 2 Conditions **eTable 5. **The Performance of Cox Regression Model Based on Stepwise Selection **eFigure 1. **The Flowchart of Patient Enrollment **eFigure 2. **Calibration of the Random Survival Forest Model **eFigure 3. **Consecutive Performance of the RSF and Cox Model **eFigure 4. **Subgroup Tests for Prognostic Stratification Ability of the Random Survival Forest Model

Supplement 2.
Data Sharing Statement
