# Supplementary material for: Machine Learning–Based Prognostic Model for Patients After Lung Transplantation
Source: JAMA Netw Open. 2023 May 5;6(5):e2312022. doi: 10.1001/jamanetworkopen.2023.12022 (PMC10163387; doi:10.1001/jamanetworkopen.2023.12022)
Supplement: Supplement 1. — eMethods. Additional Information About Data Collection, Model Development, and Model Validation eTable 1. Overall Survival Rate for Patients After Lung Transplantation eTable 2. Predicted Value by RSF Model in Patients With Different Survival Statuses eTable 3. Subgroup Tests for the RSF Model eTable 4. The Performance of RSF Model in 2 Conditions eTable 5. The Performance of Cox Regression Model Based on Stepwise Selection eFigure 1. The Flowchart of Patient Enrollment eFigure 2. Calibration of the Random Survival Forest Model eFigure 3. Consecutive Performance of the RSF and Cox Model eFigure 4. Subgroup Tests for Prognostic Stratification Ability of the Random Survival Forest Model [file jamanetwopen-e2312022-s001.pdf]

Supplemental Online Content

Tian D, Yan HJ, Huang H, et al. Machine learning–based prognostic model for patients after lung transplantation. *JAMA Netw Open*. 2023;6(5):e2312022.
doi:10.1001/jamanetworkopen.2023.12022

eMethods. Additional Information About Data Collection, Model Development, and Model Validation
eTable 1. Overall Survival Rate for Patients After Lung Transplantation
eTable 2. Predicted Value by RSF Model in Patients With Different Survival Statuses
eTable 3. Subgroup Tests for the RSF Model
eTable 4. The Performance of RSF Model in 2 Conditions
eTable 5. The Performance of Cox Regression Model Based on Stepwise Selection
eFigure 1. The Flowchart of Patient Enrollment
eFigure 2. Calibration of the Random Survival Forest Model
eFigure 3. Consecutive Performance of the RSF and Cox Model
eFigure 4. Subgroup Tests for Prognostic Stratification Ability of the Random Survival Forest Model

This supplemental material has been provided by the authors to give readers additional information about their work.

© 2023 Tian D et al. *JAMA Network Open.*

eMethods. Additional Information About Data Collection, Model Development, and Model Validation

Data Collection

A total of 22 characteristics, consisting of 4 recipient factors, 1 donor factor, 4 transplant procedural factors, and 13 posttransplant factors, were collected as follows: age, sex, body mass index (BMI), diagnosis, donor arterial oxygen tension/inspired oxygen fraction (PaO2/FiO2), surgical type, surgical approach, operation time, cold-ischemia time, intensive care unit (ICU) stay, extracorporeal membrane oxygenation (ECMO) type, postoperative ECMO time, preoperative hormone use, grade 3 primary graft dysfunction at 72 h (72 h PGD3), postoperative ventilator time, multidrug-resistant bacterial infection, 6-minute walking test (6MWT), forced expiratory volume at the first second (FEV1), FEV1 percent predicted (FEV1%), forced vital capacity (FVC), FVC percent predicted (FVC%), and FEV1/FVC. Our center prefers a double lung transplant for patients with pulmonary infection and severe pulmonary hypertension. The ECMO type consists of two categories: veno-venous cannulation ECMO and veno-arterial cannulation ECMO. Veno-veno-arterial cannulation ECMO is a variant of veno-arterial ECMO and is categorized as such. The maintenance hormone treatment is preoperatively applied to patients according to the original disease condition. MRBI is defined as a bacterial infection that is resistant to multiple antibiotics and causes symptoms. The 6MWT and pulmonary function data were collected from the first examination within 6 months post-transplantly.

Model Development

The grid search method was used for hyperparameter tuning (including number of trees, number of variables to possibly split at each node, and minimum size of terminal node). The bootstrapping resampling method was used to estimate the performance of models fitted by each parameter combination.

Model Validation

The integrated area under the curve (iAUC) of the time-dependent receiver operator characteristic curve (ROC) was used to evaluate the continuous model's discrimination ability. The higher the iAUC of a model, the better its performance in discriminating patients with different outcome statuses. The integrated Brier score (iBS) was applied to estimate the continuous calibration ability of the models. The iBS ranges from 0 to 1, and an iBS of prediction model close to 0 indicates excellent calibration. The iAUC and iBS were estimated from 1 to 48 months at 1-month intervals. Model performance at specific time points (1 month and 1 year) was assessed by the time-dependent area under the curve (tAUC) and prediction error (PE). Meanwhile, the calibration was visualized as a curve between observed survival and predicted survival.

© 2023 Tian D et al. *JAMA Network Open.*

eTable 1. Overall Survival Rate for Patients After Lung Transplantation

Survival rates [a]	All patients	Conditional on survival to 1 month	Conditional on survival to 1 year
30-day mortality	21.6%	-	-
1-year survival	64.9%	82.8%	-
3-year survival	55.5%	70.8%	85.5%
5-year survival	48.8%	62.3%	75.2%

[a] Survival rates were estimated by the Kaplan-Meier method.

© 2023 Tian D et al. *JAMA Network Open.*

eTable 2. Predicted Value by RSF Model in Patients With Different Survival Statuses

	Mean	SD	Median	IR	*P* value
**1-month survival statuses**					
**Survival**	25.93	19.98	18.94	11.23, 37.01	*P* < 0.001
**Death**	55.54	17.57	58.83	42.58, 69.71	Ref
**1-year survival statuses**					
**Survival**	20.84	15.58	16.30	10.32, 29.37	*P* < 0.001
**Death**	55.45	17.33	58.97	44.03, 69.48	Ref

Abbreviations: SD, standard deviation; IR, interquartile range.

© 2023 Tian D et al. *JAMA Network Open.*

eTable 3. Subgroup Tests for the RSF Model

Subgroups	iAUC (95% CI)	iBS (95% CI)
SLTx recipients	0.861 (0.773, 0.930)	0.159 (0.118, 0.193)
DLTx recipients	0.896 (0.837, 0.941)	0.096 (0.071, 0.125)
IPF patients	0.885 (0.817, 0.939)	0.143 (0.111, 0.178)
COPD patients	0.809 (0.606, 0.928)	0.150 (0.084, 0.201)

Abbreviations: iAUC, integrated area under the curve; iBS, integrated brier score.

© 2023 Tian D et al. *JAMA Network Open.*

eTable 4. The Performance of RSF Model in 2 Conditions

Models	Time of prediction	Selected variables	iAUC	iBS
RSF model (excluded lung function test and 6MWT data)	1 to 48 months	ICU stay, Postoperative ventilator time, Postoperative ECMO time, Age, Operation time, 72h PGD 3, Donor PaO_2/FiO2, BMI, Diagnosis, Cold ischemia time, Surgical approach, Multidrug-resistant bacteria infection	0.800 (0.693, 0.856)	0.173 (0.151, 0.196)
RSF model (excluded patients dead within 1 month)	3 to 48 months	Postoperative ventilator time, ICU stay, 6MWT, FVC%, Postoperative ECMO time, FEV1%, FEV1/FVC, FEV1, FVC, Age, Operation time, BMI, Donor PaO_2/FiO2, Diagnosis, Surgical approach, Cold ischemia time, ECMO type	0.834 (0.752, 0.897)	0.143 (0.114, 0.174)

Abbreviations: RSF, random survival forests; ICU, intensive care unit; ECMO, extracorporeal membrane oxygenation; 72 h PGD3, grade 3 primary graft dysfunction at 72h; PaO2/FiO2, arterial oxygen tension/inspired oxygen fraction; BMI, body mass index; 6MWT, 6-minute walking test; FEV1, forced expiratory volume in 1 second; FEV1%, percentage of predicted forced expiratory volume in 1-second value; FVC, forced vital capacity; FVC%, percentage of predicted forced vital capacity value; iAUC, integrated area under the curve; iBS, integrated brier score.

© 2023 Tian D et al. *JAMA Network Open.*

eTable 5. The Performance of Cox Regression Model Based on Stepwise Selection

Selected variables [a]	iAUC	iBS	*P* value [b]
Age, Diagnosis, Surgical approach, Operation time, Postoperative ECMO time, ICU stay, FEV1, FVC, FVC%, 72h PGD 3, Donor PaO_2/FiO_2	0.662	0.201	<0.001

Abbreviations: iAUC, integrated area under the curve; iBS, integrated brier score.

[a] The stepwise selection determined 11 factors to develop this Cox regression model.

[b] Comparison with the performance of Cox model to RSF model with the same time of prediction.

© 2023 Tian D et al. *JAMA Network Open.*

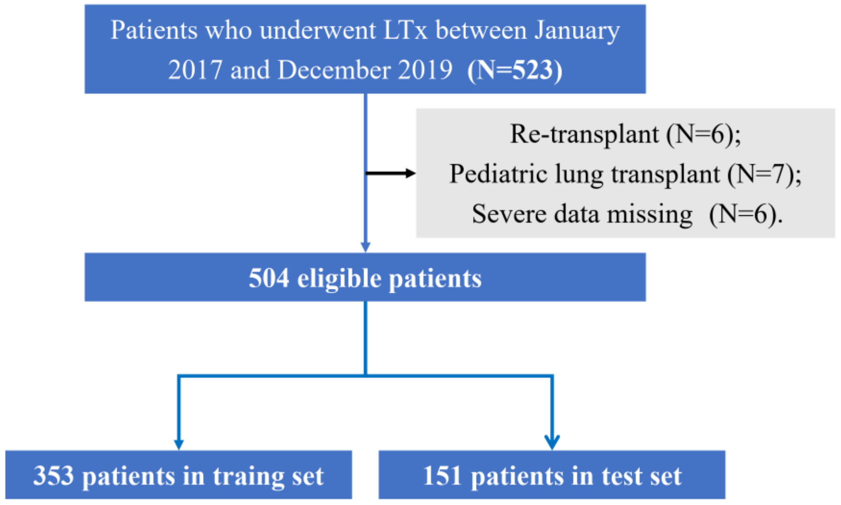

eFigure 1. The Flowchart of Patient Enrollment

Out of 523 patients after LTx, 6 patients with re-transplant, 7 with a pediatric lung transplant, and 6 with severe data missing were excluded from this study. Eventually, 504 patients were eligible and included in the analysis.

© 2023 Tian D et al. *JAMA Network Open.*

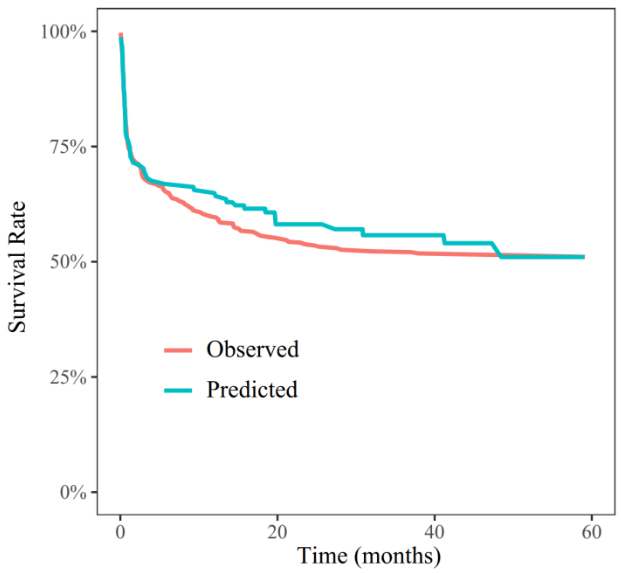

eFigure 2. Calibration of the Random Survival Forest Model

The predicted survival rate of the random survival forest model showed an excellent agreement with the observed survival rate.

© 2023 Tian D et al. *JAMA Network Open.*

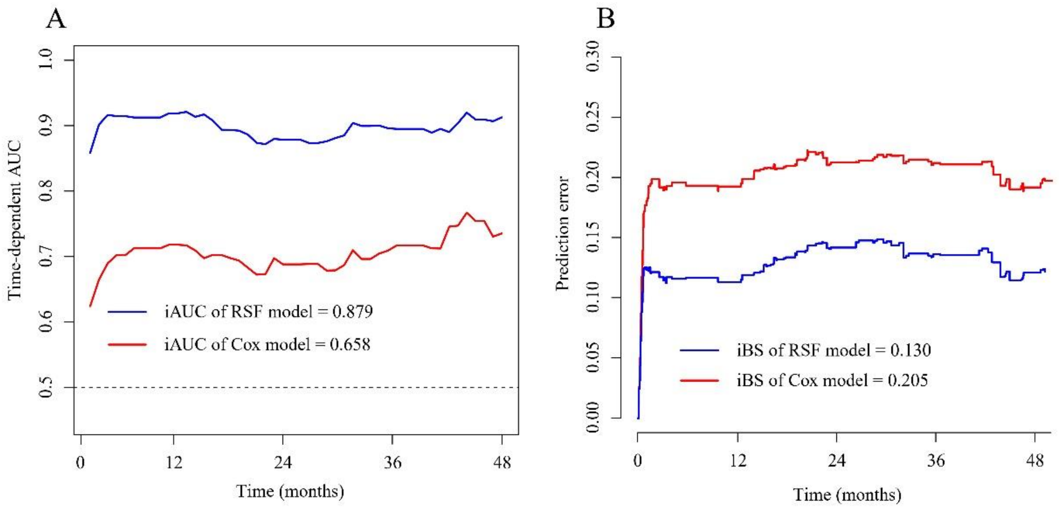

eFigure 3. Consecutive Performance of the RSF and Cox Model

Regarding discrimination (A) or calibration (B), the RSF model is superior to the Cox model from 1 month to 48 months.

Abbreviations: iAUC, integrated area under the curve; RSF, random survival forests; iBS, integrated brier score.

© 2023 Tian D et al. *JAMA Network Open.*

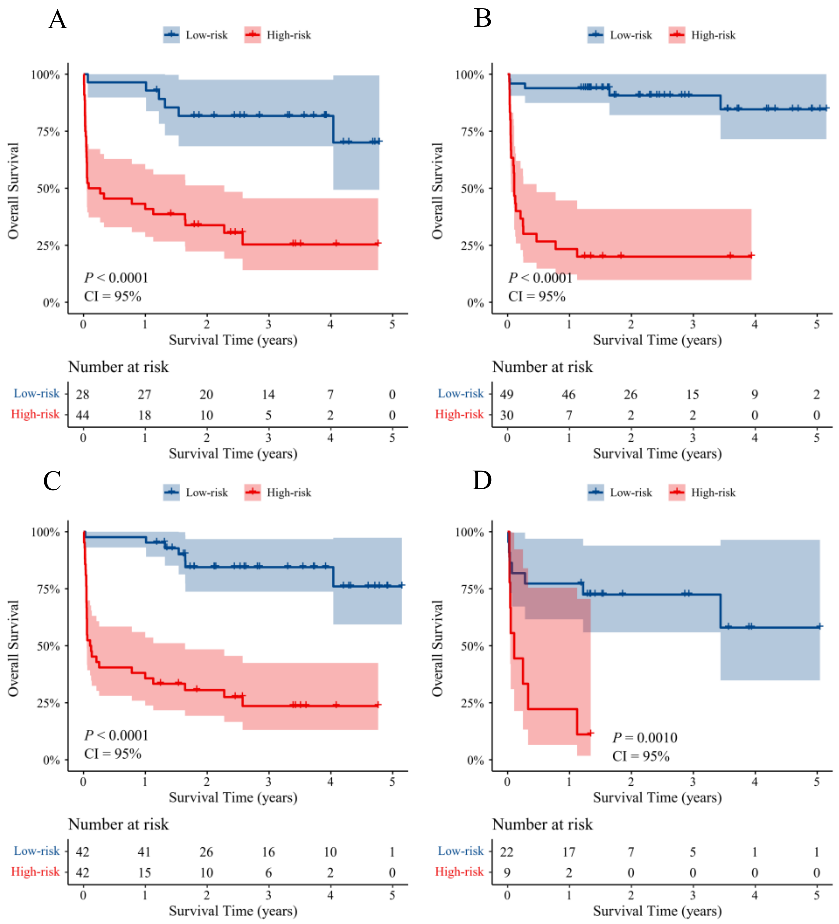

eFigure 4. Subgroup Tests for Prognostic Stratification Ability of the Random Survival Forest Model

Within the test set, the random survival forest model divided patients with single lung transplantation (A), double lung transplantation (B), interstitial pulmonary fibrosis (C), and chronic obstructive pulmonary diseases (D) to low- or high-risk of poor survival with significant differences (all $P < 0.05$).

© 2023 Tian D et al. *JAMA Network Open.*
