# Supplementary material for: Machine Learning–Based Prognostic Model for Patients After Lung Transplantation
Source: JAMA Netw Open. 2023 May 5;6(5):e2312022. doi: 10.1001/jamanetworkopen.2023.12022 (PMC10163387; doi:10.1001/jamanetworkopen.2023.12022)
Supplement: Supplement 2. — Data Sharing Statement [file jamanetwopen-e2312022-s002.pdf]

Data Sharing Statement

Tian. Machine Learning-Based Prognostic Model for Patients After Lung Transplantation. *JAMA Netw Open*. Published May 05, 2023. doi:10.1001/jamanetworkopen.2023.12022

Data
Data available: Yes
Data types: Deidentified participant data
How to access data: We will provide deidentified data, if individual request it. Email: chenjy@wuxiph.com or shilingzhi1979@126.com.
When available: With publication

Supporting Documents
Document types: Statistical/analytic code
How to access documents: We will provide deidentified data, if individual request it. Email: chenjy@wuxiph.com or shilingzhi1979@126.com.
When available: With publication

Additional Information
Who can access the data: Our data will be available to researchers whose proposed use of the data has been approved.
Types of analyses: Our data will be available for the purpose of performing a pooled analysis.
Mechanisms of data availability: Our data would be available if the requester signed a data access agreement.
